# Iron Deficiency in Menstruating Adult Women: Much More than Anemia

**DOI:** 10.1089/whr.2019.0011

**Published:** 2020-01-29

**Authors:** M. Cristina Fernandez-Jimenez, Gemma Moreno, Ione Wright, Pei-Chun Shih, M. Pilar Vaquero, Angel F. Remacha

**Affiliations:** ^1^ Hematology Department, Complejo Hospitalario de Toledo, Toledo, Spain.; ^2^ Hematology Department, Hospital Ramón y Cajal, Madrid, Spain.; ^3^ Department of Metabolism and Nutrition, Institute of Food Science, Technology and Nutrition (ICTAN), Consejo Superior de Investigaciones Científicas (CSIC), Madrid, Spain.; ^4^ Faculty of Psychology, Universidad Autónoma de Madrid, Madrid, Spain.; ^5^ Hematology Laboratory Department, Hospital Sant Pau, Barcelona, Spain.

**Keywords:** iron deficiency anemia, nonhematological symptoms, cognitive function, menstruation, women

## Abstract

***Background:*** Iron deficiency anemia (IDA) is highly prevalent in women of child-bearing age. However, their nonhematological symptoms have been overlooked. This study aims to analyze the nonhematological features and symptoms of IDA in a group of women of reproductive age and the changes occurred during iron therapy.

***Materials and Methods:*** IDA women underwent dietary, physical activity, menstrual blood loss, and cognitive function assessment at baseline. Hematological and biochemical parameters were analyzed. Executive attention was tested by the flanker task and working memory by the 2-back task. Oral iron therapy (ferrous sulfate) was given to 35 women for 8 weeks and the changes in iron status, biochemical markers, cognitive function, and nonhematological symptoms were evaluated.

***Results:*** Patients presented nonhematological symptoms: pica, 32.4%; cheilitis, 20.6%; restless legs syndrome (RLS), 20.6%; diffuse hair loss, 55.9%; and ungual alterations, 38.2%. Two or more symptoms were present in 58.8% of women. Serum iron and working memory were correlated at baseline. Multivariate analyses show associations (odds ratio [OR], 95% confidence interval [CI]) between pica and reaction time in the working memory test (OR 2.14, 95% CI 1.19–3.87, *p* = 0.012); RLS with total serum protein (OR 0.08, 95% CI 0.06–0.92, *p* = 0.043); and cheilitis with mean corpuscular hemoglobin (OR 0.388, 95% CI 0.189–0.799, *p* = 0.01). Pica, cheilitis, and RLS completely resolved with iron therapy, and ungual alterations and hair loss improved in 92.3% and 84.2% of women, respectively. Better performance in executive attention and working memory was observed after iron therapy.

***Conclusions:*** More attention should be given to the nonhematological manifestations of IDA to improve the quality of life of menstruating women.

## Introduction

Iron deficiency (ID) is the most common nutritional deficiency in the world and is the leading cause of anemia in both developed and developing countries. The greatest prevalence of iron deficiency anemia (IDA) is found in women of reproductive age and preschool children, regardless of geographic region or economic status.^1^

ID occurs as a spectrum beginning with tissue iron store depletion and progressing to impaired erythropoiesis and anemia. Iron is an essential element required for numerous vital processes, including energy metabolism, cell signaling, gene expression, and cell growth regulation and differentiation.^2^ These functions are so basic that ID has far reaching consequences on systemic functions apart from the well-known feature of anemia, but the nonhematological symptoms of this trace element deficiency have been often overlooked.

Nonhematological manifestations of ID include fatigue, reduced physical endurance, defective structure or function of epithelial tissues, pica, restless legs syndrome (RLS), decreased cognitive performance, and behavioral disturbances.^3,4^ ID impairs the health and well-being of women and increases the risk of adverse maternal and neonatal outcomes.^5,6^ Our research group has observed elevated bone remodeling in IDA young adult women that improved during iron recovery, suggesting that chronic ID may be a risk factor for future osteoporosis in women.^7–9^ According to the Global Burden of Disease Study 2016, IDA is the first cause of years lived with disability burden in women.^10^ Nevertheless, there remains a lack of awareness of the clinical consequences of IDA among this population.

The aim of this study was to prospectively analyze the characteristics, analytical data, and nonhematological signs and symptoms of IDA in a homogeneous group of women of reproductive age and their response to iron therapy.

## Materials and Methods

### Subjects

Participants were required to be Caucasian women aged between 18 and 40 years with IDA. Patients were included when they had hemoglobin ≤110 g/L, mean corpuscular volume <98 fL, transferrin saturation <15% and ferritin <20 ng/mL, or if ferritin was 20–50 ng/mL, soluble transferrin receptor (sTfR) was >5 mg/L.

Exclusion criteria were as follows: smoking, amenorrhea, menopause, current pregnancy or pregnancy within the previous year, breastfeeding, thalassemia, iron metabolism-related diseases such as hemochromatosis, bleeding disorders, autoimmune diseases, chronic gastrointestinal diseases (inflammatory bowel disease, gastric ulcers, coeliac disease, and Crohn's disease), neoplastic diseases, renal disease or hormone-related diseases independent of IDA, chronic inflammation (C-reactive protein >5 mg/L), creatinine levels >1.0 mg/dL (120 μmol/L), abnormal liver tests (>2 times normal), blood donation in the past 3 months, current use of iron supplements, and excess alcohol consumption or use of recreational drugs, prescription drugs, or herbal preparations that could interfere with iron absorption and/or affect mental performance.

Over 18 months, 84 women who met the inclusion criteria were interviewed by telephone to assess all exclusion criteria. After the exclusion criteria were applied, 36 women agreed to participate: 1 subject abandoned the study due to a change of residence.

### Study protocol

This prospective interventional study was conducted at the Complejo Hospitalario de Toledo, Spain, in 2014, as a part of a larger long-term project. The study followed guidelines stated in the Declaration of Helsinki and was approved by the Complejo Hospitalario de Toledo Clinical Research Ethics Committee and the Spanish National Research Council Ethics Committee (Bioethics subcommittee). Written informed consent was obtained from all participants before study commencement.

At enrolment, a full medical history was recorded, and anthropometric measurements were carried out. Patients underwent dietary, physical activity, menstrual blood loss, and cognitive function assessment. Blood samples were taken for complete blood count (CBC), coagulation tests, and measures of iron status and other biochemical parameters.

Patients were prescribed 8 weeks of oral iron therapy in the form of ferrous sulfate tablets (Tardyferon; Pierre Fabre Médicament, Boulogne, France): one tablet (80 mg Fe) daily if hemoglobin >100 g/L, or two tablets a day (160 mg Fe) if hemoglobin <100 g/L. Patients were instructed to take the tablets in fasting conditions with water or orange juice. At the time the study was conducted, this was the conventional treatment for ID, but mounting evidence indicates that low doses and treatment on alternate days are more effective and better tolerated.^11–13^

**FIG. 1.**
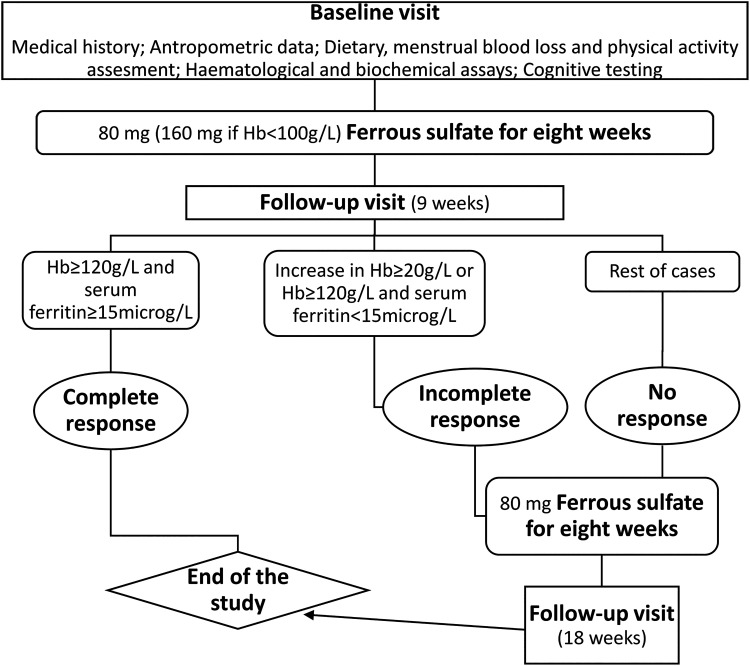
Flow chart.

A follow-up visit was scheduled 9 weeks after the baseline visit, 1 week after completing the prescribed pharmacological treatment to allow iron levels to stabilize. Blood samples were taken to repeat the CBC and biochemical parameters measurement and cognitive function was assessed again. Patients were asked to report possible side effects associated with the treatment and any health problems from their last visit. If patients had not recovered from anemia and/or ID after the first 8-week cycle of treatment (hemoglobin >120 g/L and ferritin ≥15 ng/mL), a further 8 weeks of treatment was prescribed, with a second follow-up visit scheduled 1 week after finishing the second cycle of treatment. Figure 1 shows the study flow chart.

Complete response was defined by normalization of Hb (Hb >120 g/L) and serum ferritin ≥15 ng/mL, the ferritin cutoff value suggested by the World Health Organization to indicate depleted iron stores for individuals of 5 years of age or older.^14^ A final Hb >120 g/L but serum ferritin <15 μg/L, or an increase in Hb ≥20 g/L were regarded as incomplete response.

The compliance of the study was assessed by questionnaires and a personal interview in each visit. Patients were asked about the number of tablets that were taken throughout treatment.

### Data collection

#### Clinical data

Medical history was taken by an hematologist at the time of initial evaluation through a prestructured questionnaire that included medication, dietary supplements, any past or present prestructured questionnaire medical conditions, previous operations, and reproductive history. Particular attention was given to a family history of anemia, hypercholesterolemia, hypertension, osteoporosis, and kidney or thyroid disease. Patients were questioned specifically about whether they had pica or other symptoms attributed to ID, such as fatigue, epithelial manifestations (hair loss, angular cheilitis, and brittle nails), and neuromuscular symptoms (RLS). Pica was defined as the compulsive eating of food or nonfood items, singly or in combination, not part of the patient's habitual diet or preferences.

Menstrual cycle duration, period length, and number of days with intense menstrual blood loss (heavy bleeding days) were monitored using a self-administered paper questionnaire, as described previously.^15,16^ Questions on the presence of blood clots, bleeding lasting >7 days, and whether multiple pads or tampons were saturated during the menstrual flow period were also included. Patients with heavy blood loss were voluntarily referred to a gynecologist for further evaluation. The use of oral contraceptives was also recorded.

A short questionnaire was used to assess overall physical activity. Questions included duration and intensity of daily walking and moderate- and vigorous-intensity activities per week.^17^

#### Anthropometric data

Height and weight were obtained at baseline and weight measurements were repeated on next visit/s. Weight was measured to the nearest 100 g using a medical weighing scale (Seca Ltd, Hamburg, Germany) and height was measured with a stadiometer incorporated into the scale. Body mass index (BMI) was worked out *via* the formula: weight (kg)/height (m^2^).

### Hematological and biochemical assays

Blood samples were collected between 8:00 and 10:00 by venepuncture after a 12-hour fasting period. Serum and plasma were obtained after centrifugation (for 5 minutes at 1000 *g*). The CBC was measured in whole blood following standard laboratory techniques using the Beckman Coulter LH780 Analyzer (Beckman Coulter, Brea, CA). Routine coagulation tests including prothrombin time, activated partial thromboplastin time, and fibrinogen were assayed on a blood coagulation analyzer ACL TOP 700 (Beckman Coulter). Serum iron, serum ferritin, total iron binding capacity, serum vitamin B_12_, serum folate, sTfR, and other biochemical variables were determined by modular analyzers (Elecsys and Modular DP; Roche Diagnostics, Mannheim, Germany).

### Dietary assessment

Each subject's dietary intake was evaluated at baseline with a 72-hour detailed dietary intake report, previously validated and proved valuable to assess nutrient intake^18^ specifying the types of food consumed and serving weights. The possible food intake changes throughout the study were monitored using a food frequency questionnaire. The options of frequency of consumption and their corresponding codes were as follows: never (0), less than once a week (0.5), once a week (1), two to three times a week, four to six times a week (2.5), daily (7), and more than once a day (10).

### Cognitive testing

Cognitive performance was assessed at baseline and follow-up. Two cognitive tasks were included in this study: Verbal version of the flanker task^19^ that tests executive attention and 2-back task^20^ that is widely used to measure working memory. The computerized programs of both tasks were taken from the COG-LAB-UAM Battery.^21^ Verbal flanker task required deciding, as fast as possible, whether the letter in the center of a set of three letters was vowel or consonant. The target (*e.g.*, vowel) could be surrounded by compatible (*e.g.*, vowel) or incompatible letters (*e.g.*, consonant). There were a total of 22 practice trials and 80 test trials. Half of the trials were compatible, and they were randomly presented across the session. Mean reaction time for correct responses and number of hits and errors were recorded. Given that speed–accuracy trade-off could vary between experiments, participants, and conditions, we used the inverse efficiency score (IES),^22^ which adjusts the reaction time by the proportion of correct answers, combining speed and accuracy into a single measure. High IES values indicate a less efficient performance, that is, greater difficulties in solving the task. In the 2-back task, upper and lower case letters were presented in one of eight equidistant spatial locations around the center of the screen. Stimuli were presented for 200 ms and 1300 ms were given for responding. There were 75 test stimuli of which 24 were match stimuli. Participants pressed the space bar of the keyboard to make a match response (a letter presented in the same spatial location two positions back in the sequence).

To assess potential confounding variables, subjects filled out a short questionnaire aimed to determine patients' latest education level, profession, urban–rural residence, and level of interaction with computers, before completing the computerized cognitive tests. All sessions were monitored by trained research staff, overhead lighting was kept low, the subject was asked to maintain a fixed distance from the screen, and environmental distractions were minimized.

### Statistical analysis

The collected data were analyzed using the SPSS for Windows program (version 25.0; IBM Corp., Armonk, NY). All continuous variables were analyzed for normality and, when required, were log-transformed to achieve normality. Pearson correlation coefficient was used to analyze correlations between continuous variables. A stepwise-forward multiple linear regression analysis was used to determine which variables contribute significantly to the results of cognitive tests. To identify factors associated with nonhematological symptomatology, univariate and multivariate logistic regression analyses were applied. Pretreatment and post-treatment cognitive assessments were compared using paired *t*-tests. The level of statistical significance was considered as *p* < 0.05.

## Results

### Patient characteristics and evaluation

A total of 35 anemic young women completed the study. In total, 74.3% of participants reported past personal history of IDA and 57.1% had female family history of IDA (first- and/or second-grade relatives). Patient characteristics and hematological data at baseline are summarized in Table 1.

**Table 1.** Patient Characteristics and Hematological Data at Baseline

	Mean	SD
Age	35.0	5.5
Body mass index	26.1	4.6
Energy intake (kcal/day)	2194	415
Dietary iron intake (mg/day)	16.5	5.7
Hemoglobin (g/dL)	9.99	0.97
Hematocrit (%)	31.14	2.46
Mean corpuscular volume (fL)	73.11	6.72
Mean corpuscular hemoglobin concentration (g/dL)	32.03	0.97
Red blood cell distribution width (%)	17.51	1.80
Serum ferritin (ng/mL)	4.23	1.96
Transferrin (mg/dL)	381.1	46.9
Serum iron (μg/dL)	24.83	7.87
PTH (pg/mL)	39.29	14.12

	Median	IQR
Age at menarche (years)	13	3
Menstrual cycle length (days)	28	5
Period length (days)	5	3
Heavy bleeding (days)	2	1

Values of the women who completed the study (*n* = 35).

IQR, interquartile range; PTH, parathyroid hormone; SD, standard deviation.

Most participants (85.3%) reported IDA-related nonhematological symptomatology: 32.4% pica (ice or one particular food, especially crunchy foods), 20.6% cheilitis, 20.6% RLS, 55.9% diffuse hair loss, 38.2% ungual changes, and 20.6% intense fatigue. Four patients reported headache, one tinnitus and another one dysphagia (without esophageal webs). In total, 58.8% of women had two or more symptoms associated with IDA.

Heavy menstrual bleeding according to the questionnaire was reported by 24 patients (68.6%), 9 out of them presented some physical condition affecting the uterus, such as fibroids and polyps, after further evaluation. Seven women refused gynecological examination. All patients showed normal coagulation tests. Only four women used oral contraceptives.

No significant differences were found in nutrient intake between patients nor in women's diet among baseline and end of treatment, except for “fruit juices at breakfast” that showed an increased consumption (median increased from never to once a week) (*p* = 0.015) and “other dairy products at breakfast” that decreased (median values lower than once a week) (*p* = 0.022).

Most patients reported low physical activity and none high-intensity exercise. BMI was 26.1 ± 4.6 kg/m^2^ at baseline and did not significantly vary throughout study. In total, 50% of the patients were overweight and three out of the patients had grade 1 obesity (BMI >30 kg/m^2^).

Significant associations between parameters at baseline are presented in Tables 2 and 3. Interestingly, pica, RLS, and ungual changes were related to age of menarche. In addition, associations between cognitive function measures and neurological symptoms, specifically pica and reaction time in 2-back test, were observed.

**Table 2.** Univariate and Multivariate Logistic Regression Analyses of Several Clinical and Biochemical Variables Associated with Nonhematological Symptomatology at Baseline

Symptom	Variables	Univariate		Multivariate	
		OR (95% CI)	*p*	OR (95% CI)	*p*
Pica	Menarche age	1.84 (1.05–3.22)	0.034	—	—
	Reaction time (2-back)	1.6 (1.06–2.45)	0.027	2.14 (1.19–3.87)	0.012
RLS	Menarche age	0.57 (0.32–0.99)	0.045	—	—
	Serum total protein	0.07 (0.005–0.82)	0.035	0.08 (0.06–0.92)	0.043
	Platelet count	1.03 (1.03–1.04)	0.023	—	—
Cheilitis	Mean corpuscular volume	0.64 (0.46–0.9)	0.01	—	—
	Mean corpuscular hemoglobin	0.37 (0.18–0.78)	0.008	0.388 (0.189–0.799)	0.01
	Mean corpuscular hemoglobin concentration	0.26 (0.09–0.79)	0.017	—	—
	Red blood cell distribution width	1.79 (1.02–3.13)	0.041	—	—
	Serum iron	0.7 (0.52–0.96)	0.027	—	—
	Soluble transferrin receptor	1.35 (1.02–1.78)	0.038	—	—
	Transferrin saturation	0.28 (0.08–0.9)	0.033	—	—
	Serum folate	0.51 (0.28–0.95)	0.034	—	—
Diffuse hair loss	Procollagen type 1N-terminal propeptide	1.06 (1.01–1.13)	0.045	NA	—
Ungual changes	Menarche age	0.62 (0.39–0.9)	0.045	NA	

CI, confidence interval; RLS, restless leg syndrome; NA, nonapplicable; OR, odds ratio.

**Table 3.** Associations Between Cognitive Function Measures and Other Parameters at Baseline

Test	Variable	Correlations	Multiple regression analysis *β* coefficient (SE), *p*
Flanker task	Inverse efficiency score for compatible trials	PTH: *r* = 0.43, *p* = 0.015 Serum albumin level: *r* = −0.47, *p* = 0.019 Reticulocyte count: *r* = −0.44, *p* = 0.019 Fasting glucose: *r* = 0.41, *p* = 0.025	PTH β: 0.36 (1.4), *p* = 0.035 Fasting glucose β: 0.57 (3.5), *p* = 0.002 (*R*^2^ = 0.63, *p* < 0.001)
	Inverse efficiency score for incompatible trials	PTH: *r* = 0.4, *p* = 0.041 Serum iron levels: *r* = −0.42, *p* = 0.016	PTH β: 0.52 (1.4), *p* = 0.003 (*R*^2^ = 0.52, *p* = 0.003)
2-Back test	Reaction time	Transferrin saturation: *r* = −0.37, *p* = 0.041 Fasting glucose: *r* = 0.42, *p* = 0.025	No variables were entered into the equation
	Correct responses	BMI: *r* = −0.37, *p* = 0.043 Transferrin saturation: *r* = 0.4, *p* = 0.027 Serum iron levels: *r* = 0.43, *p* = 0.018	Serum iron levels β: 0.44 (0.45), *p* = 0.01 BMI β: −0.37 (0.93), *p* = 0.029 (*R*^2^ = 0.32, *p* = 0.006)
	Omission errors	BMI: *r* = 0.37, *p* = 0.043 Serum protein level: *r* = −0.45, *p* = 0.025 Serum albumin level: *r* = −0.42, *p* = 0.047 Fasting glucose: *r* = 0.4, *p* = 0.03 Transferrin saturation: *r* = −0.34, *p* = 0.027 Serum iron levels: *r* = −0.43, *p* = 0.018	Serum iron levels β: −0.4 (0.39), *p* = 0.018 BMI β: 0.37 (0.8), *p* = 0.037 Fasting glucose β: 0.49 (0.58), *p* = 0.007 (*R*^2^ = 0.54, *p* < 0.001)
	Commission errors	Significant correlations not found	

BMI, body mass index; SE, standard error.

### Outcome after iron therapy

Thirty-four out of 35 patients showed response to oral iron treatment, 24 showed complete response (16 at 8 weeks and 8 at 16 weeks follow-up), and 10 showed incomplete response. The patient who did not respond to therapy (refractoriness) was excluded from the study. This patient was further diagnosed with *Helicobacter pylori* infection.

Nineteen patients (54.3%) notified side effects that were gastrointestinal symptoms including nausea, constipation, loose stools, and epigastric pain. Reported side effects were all mild, transient, and self-limited and did not require discontinuation of treatment.

All cases of pica, cheilitis, and RLS completely resolved with iron therapy, even when some patients did not achieve complete response to treatment. Ungual changes and hair loss improved in 92.3% and 84.2% of women, respectively, but were not totally resolved. Fatigue, headache, tinnitus, and dysphagia disappeared after treatment. The clinical data and response to iron treatment are summarized in Table 4.

**Table 4.** Characteristics of Nonhematological Symptoms in Patients with Iron Deficiency Anemia and Response to Therapy

Nonhematological symptoms	Patients (%)	Oral iron response (%)
Cheilitis	20.6	100
Pica	32.4	100
Restless leg syndrome	20.6	100
Hair loss	55.9	84.2
Ungual changes	38.2	92.3
Headache	11.7	100
Others	5.8	100

No. of symptoms	Patients (%)	
1	35.3	
2	26.5	
3	17.6	
4	3	
5	3	

Regarding cognitive function assessment, a better performance in executive attention and working memory was observed after iron therapy, measured by Flanker and 2-back tests, as is shown in Table 5. In the Flanker task, an improvement of the IES was seen in compatible and incompatible trials, and in the 2-back task, a decrease in response latencies and an increase in accuracy were observed.

**Table 5.** Changes in Cognitive Function Measures after Iron Therapy

Test	Variable	Baseline (mean ± SD)	End of treatment (mean ± SD)	Paired *t*-test	
				*t*	*p*
Flanker task	Inverse efficiency score for compatible trials (ms)	301.9 ± 132.5	233.5 ± 37.7	3.2	0.004
	Inverse efficiency score for incompatible trials (ms)	312.2 ± 131	253.6 ± 41.4	2.6	0.014
2-Back test	Reaction time (ms)	1333 ± 2.2	1332.4 ± 1.8	2.9	0.008
	Correct responses (%)	57.9 ± 22.5	68 ± 21.6	−2.3	0.029
	Omission errors (%)	42.1 ± 22.5	32 ± 21.6	2.3	0.029
	Commission errors (%)	15.9 ± 14.6	15 ± 14.2	−0.3	ns

Different patterns of associations among cognitive function measures and biochemical and clinical data were observed after treatment. The only correlations that remained statistically significant were between IES for incompatible trials (Flanker task) and BMI (*r* = 0.42, *p* = 0.020), and between correct responses and omission errors (2-back test) and BMI (*r* = 0.43, *p* = 0.016; *r* = 0.43, *p* = 0.015). In addition, increase in ferritin levels and reticulocyte count was associated with a reduction in omission errors (*r* = −0.40, *p* = 0.034; *r* = −0.49, *p* = 0.026) and an increment in correct responses (*r* = 0.42, *p* = 0.027; *r* = 0.50, *p* = 0.026).

Changes in biochemical parameters after iron treatment included significant increases in serum folate, vitamin B_12_, glucose, uric acid, total cholesterol, high-density lipoprotein cholesterol, and bilirubin. Urea decreased significantly. These results have been discussed elsewhere.^23^

## Discussion

This research has focused on emphasizing the importance of the nonhematological manifestations of IDA in women of reproductive age and on identifying factors associated with this condition. Although several published studies have concentrated on one specific aspect such as pica and cognitive function impairment, we have tried to analyze all the data taken as a whole.

Concerning medical history, we found that more than half of our patients reported family history of IDA, which is consistent with studies suggesting that susceptibility to ID is, in part, influenced by genetics. Genetic variants of iron genes, especially TMPRSS6, HFE, transferrin, and hepcidin, may predispose individuals to IDA or protect them from it.^16,18,24,25^ In contrast, a personal history of IDA was present in almost three quarters of the patients. These findings support the convenience of screening for IDA in those with either prior personal or family histories of IDA.

As regards etiology, among women of reproductive age, menstrual blood loss is the most common cause of ID and IDA. Women with heavy menstrual bleeding are at a higher risk.^15,18,26^ In line with these data, 68.6% of the patients in our study reported heavy menstrual bleeding and few women used oral contraceptives that are known to regulate menstruation. Regarding the different factors for IDA, high menstrual blood losses and no use of oral contraceptives, but not dietary iron intake, have been consistently associated with poor iron status in women,^15,18,27,28^ emphasizing the importance of recognition and diagnosis of ID in this particularly vulnerable female population.

Most of our patients (85.3%) reported nonhematological symptoms, highlighting its high incidence and the fact that this symptomatology is often overlooked if patients are not directly questioned about it. Diffuse hair loss was the most prevalent symptom we observed (55.9% of patients) followed by brittle nails (38.2%). In the literature, both signs have been associated with chronic ID and could at least partly arise from alteration in epithelial cellular replication produced by ID.^2^ Telogen effluvium, or the rapid shift of hair from anagen (growing period) to telogen (resting period) phase, has been described as the mechanism underlying hair loss in women with ID.^29^ The most typical lesion seen in nails associated with ID is koilonychia, defined as a concavity of the outer surface of the nail but it seems that this finding is now encountered rarely, possible due to earlier detection of ID. Thinning and flattening of the nail, as well as brittle nails, precede the development of koilonychia.^30^ The fact that our patients reported improvement but not complete resolution of hair loss or brittle nails after iron administration could indicate that there are other factors involved or that a longer follow-up is necessary. In line with this, it has been reported that, even with therapy, koilonychia takes a long period of time to return to a normal appearance.^30^ Regarding hair loss, most authors recommend maintaining serum ferritin at levels >40 ng/mL,^29^ which would need longer to be achieved. Another ID-related alteration of epithelium, angular cheilitis, characterized by ulcerations or fissures at the corners of the mouth, was observed in 20.6% of patients and completely resolved with iron treatment. Angular stomatitis is not specific of ID and can also been seen in other nutritional deficiencies. It has been reported that IDA predisposes to angular cheilitis and the lesions healed with iron supplementation.^31^

IDA and ID also have a well-known association with intense fatigue,^32^ which was present in almost a quarter of the patients in our study. All cases reversed with iron therapy. It could be argued that anemia could be responsible for fatigue, but it has been observed in nonanemic individuals with ID in several reports. A recent meta-analysis of studies in iron-deficient non-anemic patients found that iron therapy improved objective and self-rated assessments of fatigue,^33^ reinforcing the fact that ID is a broader condition with extensive health consequences in addition to the well-known feature of anemia. These data highlight that for women of childbearing age with unexplained prolonged fatigue, ID should be considered.

Iron is also recognized to play a crucial role in maintenance of neuronal activity and networks.^2^ Linked to this function, two neurological symptoms, pica and RLS, have been associated with ID. Pica was reported by 32.4% of our patients, consistent with the prevalence observed in other studies.^34^ Careful inquiry was necessary because some patients were ashamed of their behavior or underrated its importance. Various forms of pica have been associated with ID, pagophagia (pica for ice) being considered quite specific.^35^ In our study, patients reported either pagophagia or food pica, a subtype of pica that consists of compulsively eating one particular food, especially if crunchy.^36^ Exact pathophysiology of pica in association with ID is unknown but it is probably attributable to ID in the central nervous system. The reasons why some patients with ID manifest pica and others do not have yet to be satisfactorily explained, but heritable traits could contribute to pica susceptibility in adults with ID. All cases of pica in this study resolved rapidly with iron therapy even before any increase was noted in the hemoglobin concentration, in agreement with previous reports.^34^

RLS is a common disorder that manifests as an intense urge to move the legs that is uncomfortable and interferes with sleep. Although the exact pathophysiology remains unclear, brain ID and altered dopaminergic function appear to play an important role in the pathogenesis of this condition.^37^ In our patients, the prevalence of clinically significant RLS was 20.6%, higher than that seen in the general population (5%–15%),^38^ and all cases resolved with iron administration. Despite its significant sleep morbidity, RLS with IDA is often overlooked. In addition, we found that patients with RLS showed lower serum protein level than the rest of IDA women, which is according to a recent study reporting lower serum albumin levels in RLS patients than in a control group.^39^

A considerable body of evidence has established that appropriate levels of brain iron are necessary for optimal brain development and functioning.^40^ Nonetheless, despite the high prevalence of ID in women of reproductive age, relatively few studies^41–45^ have examined the relationship between iron status and cognition in this group, focusing instead on infants and children. Our study found a correlation between iron serum level and working memory measured by 2-back task at baseline. After iron treatment, we detected an improvement in attention and working memory tests measured by Flanker and 2-back tasks, corroborating others' previous findings that show that cognitive alterations are responsive to iron administration.^46–48^ Of note, there was a significant decrease in omission but not commission errors in 2-back. This is in line with studies reporting that these two types of errors have different correlates and, thus, may represent different processes.^49^

In addition to correlations related to iron metabolism, we observed an intriguing association between parathyroid hormone (PTH) levels and IES (in compatible as well as in incompatible trials) in Flanker task at baseline. This finding could be related to the link between higher serum PTH levels and increased odds of poor cognition suggested by some reports, although it is not yet well established.^50^ Despite the fact our study initially seemed to corroborate this possible link, the association between PTH levels and Flanker test was not significant after iron therapy. One possible explanation could be that IDA patients presented higher PTH levels at the beginning of the trial. Linked to this, higher levels of PTH have been reported in patients with low hemoglobin and low ferritin.^51^ Nevertheless, we could not detect statistically significant differences in PTH levels before and after therapy, although the small size of our sample limits the results of our study. We also found an association between BMI and accuracy in 2-back task both at the baseline and after treatment, which agrees with the impaired working memory performance reported in overweight and obese young adults compared with healthy weight controls.^52^

## Conclusions

Despite the high prevalence of ID and IDA among women, there is insufficient awareness of its unfavorable consequences beyond anemia. Iron plays an integral role in a wide range of physiological functions; therefore, the health consequences of ID and IDA are extensive and affect all aspects of the physical health and well-being of women.

## Abbreviations Used

BMI: body mass index
CI: confidence interval
CBC: complete blood count
IES: inverse efficiency score
IQR: interquartile range
ID: iron deficiency
IDA: iron deficiency anemia
OR: odds ratio
PTH: parathyroid hormone
RLS: restless legs syndrome
SD: standard deviation
SE: standard error

## References

[1] Kassebaum NJ, Jasrasaria R, Naghavi M, et al. A systematic analysis of global anemia burden from 1990 to 2010. Blood 2014;123:615–624 24297872 10.1182/blood-2013-06-508325 PMC3907750

[2] Musallam KM, Taher AT Iron deficiency beyond erythropoiesis: Should we be concerned? Curr Med Res Opin 2018;34:81–93 29050512 10.1080/03007995.2017.1394833

[3] Lopez A, Cacoub P, Macdougall IC, Peyrin-Biroulet L Iron deficiency anaemia. Lancet (London) 2016;387:907–916 10.1016/S0140-6736(15)60865-0 26314490

[4] Pompano LM, Haas JD Increasing iron status through dietary supplementation in iron-depleted, sedentary women increases endurance performance at both near-maximal and submaximal exercise intensities. J Nutr 2019;149:231–239 30649365 10.1093/jn/nxy271

[5] Janbek J, Sarki M, Specht IO, Heitmann BL A systematic literature review of the relation between iron status/anemia in pregnancy and offspring neurodevelopment. Eur JClin Nutr 2019;73:1561–1578 30783211 10.1038/s41430-019-0400-6

[6] Friedman AJ, Chen Z, Ford P, et al. Iron deficiency anemia in women across the life span. J Womens Health (Larchmt) 2012;21:1282–1289 23210492 10.1089/jwh.2012.3713

[7] Wright I, Blanco-Rojo R, Fernandez MC, et al. Bone remodelling is reduced by recovery from iron-deficiency anaemia in premenopausal women. J Physiol Biochem 2013;69:889–896 23813442 10.1007/s13105-013-0266-3

[8] Toxqui L, Vaquero MP Chronic iron deficiency as an emerging risk factor for osteoporosis: A hypothesis. Nutrients 2015;7:2324–2344 25849944 10.3390/nu7042324 PMC4425147

[9] Toxqui L, Perez-Granados AM, Blanco-Rojo R, Wright I, de la Piedra C, Vaquero MP Low iron status as a factor of increased bone resorption and effects of an iron and vitamin D-fortified skimmed milk on bone remodelling in young Spanish women. Eur J Nutr 2014;53:441–448 23771807 10.1007/s00394-013-0544-4

[10] GBD 2016 Disease and Injury Incidence and Prevalence Collaborators. Global, regional, and national incidence, prevalence, and years lived with disability for 328 diseases and injuries for 195 countries, 1990–2016: A systematic analysis for the Global Burden of Disease Study 2016. Lancet (London) 2017;390:1211–1259 10.1016/S0140-6736(17)32154-2 PMC5605509 28919117

[11] Stoffel NU, Zeder C, Brittenham GM, Moretti D, Zimmermann MB Iron absorption from supplements is greater with alternate day than with consecutive day dosing in iron-deficient anemic women. Haematologica 2019 8 14. doi: 10.3324/haematol.2019.220830 [Epub ahead of print] PMC7193469 31413088

[12] Stoffel NU, Cercamondi CI, Brittenham G, et al. Iron absorption from oral iron supplements given on consecutive versus alternate days and as single morning doses versus twice-daily split dosing in iron-depleted women: Two open-label, randomised controlled trials. Haematology 2017;4:e524–e533 29032957 10.1016/S2352-3026(17)30182-5

[13] Moretti D, Goede JS, Zeder C, et al. Oral iron supplements increase hepcidin and decrease iron absorption from daily or twice-daily doses in iron-depleted young women. Blood 2015;126:1981–1989 26289639 10.1182/blood-2015-05-642223

[14] World Health Organization. Serum ferritin concentrations for the assessment of iron status and iron deficiency in populations. Geneva, Switzerland: Vitamin and Mineral Nutrition Information System, 2011

[15] Toxqui L, Perez-Granados AM, Blanco-Rojo R, Wright I, Vaquero MP A simple and feasible questionnaire to estimate menstrual blood loss: Relationship with hematological and gynecological parameters in young women. BMC Women's Health 2014;14:71 24886470 10.1186/1472-6874-14-71 PMC4046034

[16] Blanco-Rojo R, Baeza-Richer C, Lopez-Parra AM, et al. Four variants in transferrin and HFE genes as potential markers of iron deficiency anaemia risk: An association study in menstruating women. Nutr Metab (Lond) 2011;8:69 21978626 10.1186/1743-7075-8-69 PMC3195693

[17] Blanco-Rojo R, Perez-Granados AM, Toxqui L, Gonzalez-Vizcayno C, Delgado MA, Vaquero MP Efficacy of a microencapsulated iron pyrophosphate-fortified fruit juice: A randomised, double-blind, placebo-controlled study in Spanish iron-deficient women. Br J Nutr 2011;105:1652–1659 21303569 10.1017/S0007114510005490

[18] Blanco-Rojo R, Toxqui L, Lopez-Parra AM, et al. Influence of diet, menstruation and genetic factors on iron status: A cross-sectional study in Spanish women of childbearing age. Int J Mol Sci 2014;15:4077–4087 24663082 10.3390/ijms15034077 PMC3975385

[19] Eriksen BA, Eriksen CW Effects of noise letters upon identification of a target letter in a non-search task Percept Psychophysics 1974;16:143–149

[20] Kirchner WK. Age differences in short-term retention of rapidly changing information. J Exp Psychol 1958;55:352–358 13539317 10.1037/h0043688

[21] Shih PC, Privado J, Colom R. Cog-Lab-UAM. Poster presented at the X meeting of the Spanish Society for the Study of Individual Differences (SEIDI). Salamanca, 9 26, 2008

[22] Townsend JT, Ashby FG Methods of modeling capacity in simple processing systems. In Castellan J, Restle F (Eds.), Cognitive theory. Vol. 3 (pp. 200–239). Hillsdale, NJ: Erlbaum 1978

[23] Remacha AF, Wright I, Fernandez-Jimenez MC, et al. Vitamin B12 and folate levels increase during treatment of iron deficiency anaemia in young adult woman. Int J Lab Hematol 2015;37:641–648 25959209 10.1111/ijlh.12378

[24] Camaschella C. Iron deficiency. Blood 2019;133:30–39 30401704 10.1182/blood-2018-05-815944

[25] Sarria B, Navas-Carretero S, Lopez-Parra AM, et al. The G277S transferrin mutation does not affect iron absorption in iron deficient women. Eur J Nutr 2007;46:57–60 17206377 10.1007/s00394-006-0631-x

[26] Mirza FG, Abdul-Kadir R, Breymann C, Fraser IS, Taher A Impact and management of iron deficiency and iron deficiency anemia in women's health. Expert Rev Hematol 2018;11:727–736 30019973 10.1080/17474086.2018.1502081

[27] Gallego-Narbon A, Zapatera B, Vaquero MP Physiological and dietary determinants of iron status in Spanish vegetarians. Nutrients 2019;11:pii: 10.3390/nu11081734 PMC6723975 31357549

[28] Sekhar DL, Murray-Kolb LE, Kunselman AR, Weisman CS, Paul IM Differences in risk factors for anemia between adolescent and adult women. J Womens Health (Larchmt) 2016;25:505–513 26835887 10.1089/jwh.2015.5449 PMC4876539

[29] Almohanna HM, Ahmed AA, Tsatalis JP, Tosti A The role of vitamins and minerals in hair loss: A review. Dermatol Ther 2019;9:51–70 10.1007/s13555-018-0278-6 PMC6380979 30547302

[30] Sato S. Iron deficiency: Structural and microchemical changes in hair, nails, and skin. Sem Dermatol 1991;10:313–319 1764360

[31] Murphy NC, Bissada NF Iron deficiency: An overlooked predisposing factor in angular cheilitis. J Am Dietec Assoc (1939) 1979;99:640–641 10.14219/jada.archive.1979.0340 292723

[32] DeLoughery TG. Iron deficiency anemia. Med Clin North Am 2017;101:319–332 28189173 10.1016/j.mcna.2016.09.004

[33] Yokoi K, Konomi A Iron deficiency without anaemia is a potential cause of fatigue: Meta-analyses of randomised controlled trials and cross-sectional studies. Br J Nutr 2017;117:1422–1431 28625177 10.1017/S0007114517001349

[34] Borgna-Pignatti C, Zanella S Pica as a manifestation of iron deficiency. Expert Rev Hematol 2016;9:1075–1080 27701928 10.1080/17474086.2016.1245136

[35] Brown WD, Dyment PG Pagophagia and iron deficiency anemia in adolescent girls. Pediatrics 1972;49:766–767 4504176

[36] Crosby WH. Food pica and iron deficiency. Arch Int Med 1971;127:960–961 5284066

[37] Bollu PC, Yelam A, Thakkar MM Sleep medicine: Restless legs syndrome. Mo Med 2018;115:380–387 30228773 PMC6140269

[38] Yeh P, Walters AS, Tsuang JW Restless legs syndrome: A comprehensive overview on its epidemiology, risk factors, and treatment. Sleep Breath 2012;16:987–1007 22038683 10.1007/s11325-011-0606-x

[39] Olgun Yazar H, Yazar T, Ozdemir S, Kasko Arici Y Serum C-reactive protein/albumin ratio and restless legs syndrome. Sleep Med 2019;58:61–65 31129525 10.1016/j.sleep.2019.02.022

[40] Murray-Kolb LE. Iron and brain functions. Curr Opp Clin Nutr Met Care 2013;16:703–707 10.1097/MCO.0b013e3283653ef8 24100670

[41] Greig AJ, Patterson AJ, Collins CE, Chalmers KA Iron deficiency, cognition, mental health and fatigue in women of childbearing age: A systematic review. J Nutr Sci 2013;2:e14 25191562 10.1017/jns.2013.7 PMC4153327

[42] Lomagno KA, Hu F, Riddell LJ, et al. Increasing iron and zinc in pre-menopausal women and its effects on mood and cognition: A systematic review. Nutrients 2014;6:5117–5141 25405366 10.3390/nu6115117 PMC4245583

[43] Scott SP, Murray-Kolb LE Iron status is associated with performance on executive functioning tasks in nonanemic young women. J Nutr 2016;146:30–37 26661838 10.3945/jn.115.223586

[44] Blanton CA, Green MW, Kretsch MJ Body iron is associated with cognitive executive planning function in college women. Br J Nutr 2013;109:906–913 22676919 10.1017/S0007114512002620

[45] Wenger MJ, Rhoten SE, Murray-Kolb LE, et al. Changes in iron status are related to changes in brain activity and behavior in rwandan female university students: Results from a randomized controlled efficacy trial involving iron-biofortified beans. J Nutr 2019;149:687–697 30926992 10.1093/jn/nxy265 PMC6461719

[46] Falkingham M, Abdelhamid A, Curtis P, Fairweather-Tait S, Dye L, Hooper L The effects of oral iron supplementation on cognition in older children and adults: A systematic review and meta-analysis. Nutr J 2010;9:4 20100340 10.1186/1475-2891-9-4 PMC2831810

[47] Scott SP, Murray-Kolb LE, Wenger MJ, et al. Cognitive performance in indian school-going adolescents is positively affected by consumption of iron-biofortified pearl millet: A 6-month randomized controlled efficacy trial. J Nutr 2018;148:1462–1471 30016516 10.1093/jn/nxy113

[48] Wenger MJ, Murray-Kolb LE, Nevins JE, et al. Consumption of a double-fortified salt affects perceptual, attentional, and mnemonic functioning in women in a randomized controlled trial in India. J Nutr 2017;147:2297–2308 29021371 10.3945/jn.117.251587 PMC6519426

[49] Meule A. Reporting and interpreting working memory performance in n-back tasks. Front Psychol 2017;8:352 28326058 10.3389/fpsyg.2017.00352 PMC5339218

[50] Lourida I, Thompson-Coon J, Dickens CM, et al. Parathyroid hormone, cognitive function and dementia: A systematic review. PLoS One 2015;10:e0127574 26010883 10.1371/journal.pone.0127574 PMC4444118

[51] Atmaca M OM, Tasdemir E, Ozbay M Correlation of parathyroid hormone and hemoglobin levels in normal renal function. Acta Endo (Buc) 2011;7:317–323

[52] Dye L, Boyle NB, Champ C, Lawton C The relationship between obesity and cognitive health and decline. Proc Nutr Soc 2017;76:443–454 28889822 10.1017/S0029665117002014

